# Enzymatic activity of cGAS in the presence of three types of DNAs: limited cGAS stimulation by single-stranded HIV-1 SL2 DNA

**DOI:** 10.1042/BSR20240269

**Published:** 2024-04-04

**Authors:** Mineyuki Mizuguchi, Niko Kyan, Suzuka Nishimata, Yuko Nabeshima, Takayuki Obita

**Affiliations:** Faculty of Pharmaceutical Sciences, University of Toyama, Toyama 930-0194, Japan

**Keywords:** cGAMP, cGAS, enzymatic activity, G3 Y-from DNA, HIV-1 SL2 DNA

## Abstract

Cyclic GMP-AMP (cGAMP) synthase (cGAS) is activated by binding to DNA. Activated cGAS produces 2′3′-cGAMP, which subsequently binds to the adaptor protein STING (stimulator of interferon genes). This interaction triggers the cGAS/STING signaling pathway, leading to the production of type I interferons. Three types of DNA, namely double-stranded DNA longer than 40 base pairs, a 70-nucleotide single-stranded HIV-1 DNA known as SL2, and Y-form DNA with unpaired guanosine trimers (G3 Y-form DNA), induce interferon production by activating cGAS/STING signaling. However, the extent of cGAS activation by each specific DNA type remains unclear. The comparison of cGAS stimulation by various DNAs is crucial for understanding the mechanisms underlying cGAS-mediated type I interferon production in the innate immune response. Here, we revealed that cGAS produces 2′3′-cGAMP at a significantly lower rate in the presence of single-stranded SL2 DNA than in the presence of double-stranded DNA or G3 Y-form DNA. Furthermore, the guanine-to-cytosine mutations and the deletion of unpaired guanosine trimers significantly reduced the 2′3′-cGAMP production rate and the binding of cGAS to Y-form DNA. These studies will provide new insights into the cGAS-mediated DNA-sensing in immune response.

## Introduction

Recognition of cytosolic DNA resulting from bacterial and viral infections is crucial for the innate immune system’s ability to detect pathogens. Cytosolic DNA is recognized by pattern recognition receptors that activate downstream signaling pathways and induce the production of type I interferons [1]. One of these receptors, cyclic guanosine monophosphate (GMP)-adenosine monophosphate (AMP) synthase (cGAS), responds to pathogen infections [2]. Upon binding to cytosolic DNA, cGAS becomes activated and synthesizes 2′3′ cyclic-GMP-AMP dinucleotide (2′3′-cGAMP) from adenosine triphosphate (ATP) and guanosine triphosphate (GTP) [2–5]. The 2′3′-cGAMP molecule contains a phosphodiester linkage between the 2′-OH of GMP and the 5′-phosphate of AMP, as well as another linkage between the 3′-OH of AMP and the 5′-phosphate of GMP [3]. Importantly, 2′3′-cGAMP acts as a high-affinity ligand for the stimulator of interferon genes (STING), an endoplasmic-reticulum-resident membrane adaptor [3]. Upon binding to 2′3′-cGAMP, STING undergoes a conformational change that activates the cGAS/STING-dependent signaling pathway, ultimately leading to the production of type I interferons [2,3,6,7]. Therefore, cGAS functions as a DNA sensor in the signal transduction of the cGAS/STING pathway, which is critical for the innate immune response.

Human cGAS is a 522-amino acid protein consisting of a positively charged disordered region (residues 1-157), a nucleotidyl transferase (NTase) core scaffold (residues 160-330), and a Mab21 homology region (residues 213-513). The Mab21 region contains a unique zinc thumb (residues 389-405) that recognizes B-form double-stranded DNA [4,8–10]. The overall sequence of cGAS is needed to regulate the production of 2′3′-cGAMP: it is controlled by residues 1-146 in the disordered region, and residues 147-522, which comprise the NTase core and the Mab21 region [11]. In the case of double-stranded DNA, the affinity between cGAS and DNA increases in a sequence-independent but length-dependent manner. X-ray crystal structures have shown that cGAS binds double-stranded DNA through interactions with the sugar-phosphate backbone, forming a minimal 2:2 complex with DNA [12–14]. The DNA binding of cGAS has been shown to promote the formation of liquid-like droplets in which DNA-bound cGAS synthesizes 2′3′-cGAMP [11,15].

Various DNAs have been shown to induce interferon production by activating the cGAS/STING signaling pathway. One such stimulatory DNA for cGAS is a double-stranded DNA longer than 40 base pairs (bp). For example, double-stranded DNA induces type I interferon production in HEK 293T cells stably expressing STING [8]. Another stimulatory DNA is SL2, a 70-nucleotide single-stranded DNA that originates from the reverse transcription product of the first 181 nucleotides of the HIV-1 RNA genome (referred to as HIV sstDNA). The HIV sstDNA has three stem-loop structures (SL1, SL2, and SL3). Among them, SL2 has been shown to induce cGAS-dependent type I interferon production [16]. A third stimulatory DNA is the G3 Y-form, which consists of a 20-bp duplex region and four unpaired guanosine trimers at both the 5′ and 3′ ends. The G3 Y-form DNA has been proposed as a minimal cGAS recognition motif that enables the detection of HIV-1 single-stranded DNA [16]. These DNAs induce type I interferon production by activating the cGAS/STING signaling pathway [8,16]. However, because no study has quantitatively compared the cGAS stimulation induced by these DNAs, the extent to which each DNA activates cGAS has been unclear. The comparison of cGAS stimulation by various DNAs is crucial for understanding the mechanisms underlying cGAS-mediated type I interferon production in the innate immune response.

In the present study, we investigated the cGAS stimulation induced by a 50 bp double-stranded DNA from *Bacillus subtilis*, single-stranded SL2 DNA derived from HIV sstDNA, and G3 Y-form DNA. The cGAS stimulatory activity of single-stranded SL2 HIV-1 DNA was approximately 5% of that induced by the 50 bp double-stranded DNA from *Bacillus subtilis*. Furthermore, the guanine-to-cytosine mutations and the deletion of unpaired guanosine trimers significantly reduced the 2′3′-cGAMP production rate and the binding of cGAS to Y-form DNA. These studies will provide new insights into the cGAS-mediated DNA-sensing in immune response.

## Results

### Enzymatic activity assay of cGAS by HPLC analysis

cGAS synthesizes the nucleotide second messenger 2′3′-cGAMP from ATP and GTP upon binding to various DNAs. The DNAs that stimulate the cGAS/STING pathway include 50 bp double-stranded DNA from *Bacillus subtilis*, the 70-nucleotide single-stranded SL2 DNA derived from HIV-1, and the G3-ended Y-form DNA [8,16]. Although these DNAs have been shown to induce type I interferon production through the activation of the cGAS/STING pathway, there has not yet been a direct comparison of their cGAS stimulatory activity.

**Figure 1:**
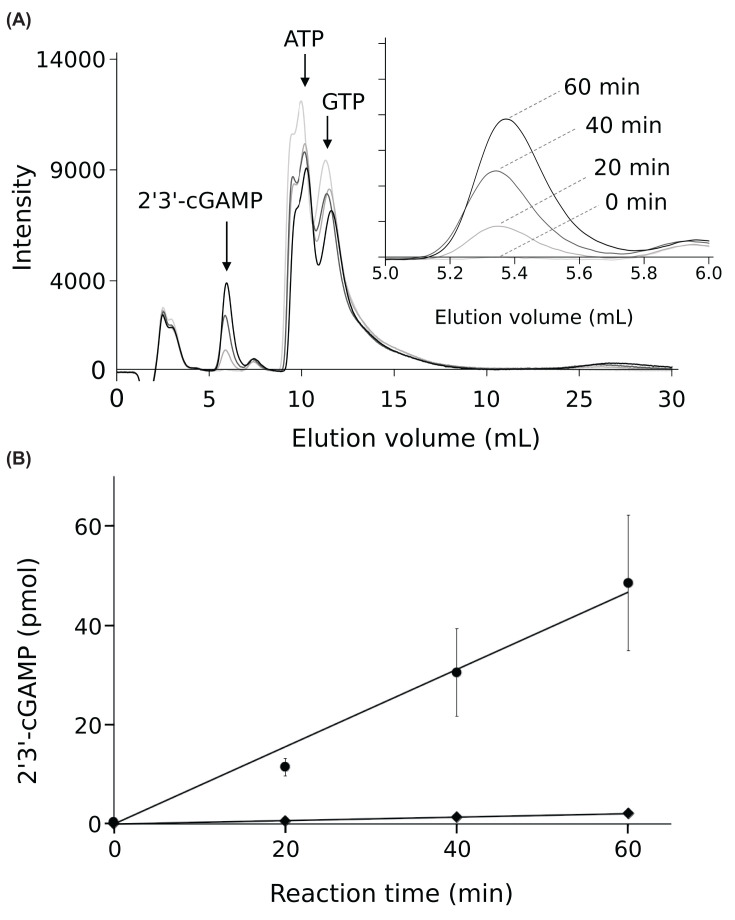
cGAS enzymatic assay using HPLC (**A**) HPLC elution profiles of samples from cGAS enzymatic assays at 0, 20, 40, and 60 min. Peaks corresponding to 2′3′-cGAMP, ATP, and GTP are indicated by arrows. The inset illustrates the 2′3′-cGAMP peaks at each time point. (**B**) Time-dependent production of 2′3′-cGAMP by cGAS. The graph illustrates the production of 2′3′-cGAMP by cGAS over time, with a comparison between reactions using 50 bp double-stranded DNA (represented by circles) and 70-nucleotide single-stranded SL2 DNA from HIV-1 (represented by diamonds). Five independent experiments were performed for 50 bp double-stranded DNA. Three independent experiments were performed for 70-nucleotide single-stranded SL2 DNA. Average and standard deviation values are shown in the graph.

To compare the levels of cGAS stimulatory activity of these DNAs, the enzymatic activity of full-length cGAS was monitored using HPLC. The enzymatic assay was conducted with an excess amount of DNA, where the DNA concentration was set to 3 μM, and the concentration of cGAS was set to 0.2 μM. The reaction mixture contained 5 mM MgCl_2_ and 0.1 mM ZnCl_2_, since Mg^2+^ and Zn^2+^ enhance cGAS activity [11,17]. Previous study has shown that DNA-activated cGAS utilizes Mg^2+^ as its catalytic cofactor [17]. The cGAS-DNA complex is stabilized by the binding of Zn^2+^ in the zinc thumb, which recognizes B-form DNA [8,9]. 2′3′-cGAMP was detected and separated from ATP and GTP using HPLC with an isocratic elution mode. The peaks of 2′3′-cGAMP appeared as the enzyme reaction proceeded (Figure 1A). The amounts of 2′3′-cGAMP were plotted against the reaction time to determine the production rate (*V*_p_) of 2′3′-cGAMP (Figure 1B).

### The cGAS stimulatory activity of 70-nucleotide single-stranded SL2 DNA from HIV-1 is extremely low compared with that of double-stranded DNA

The 2′3′-cGAMP production rate by cGAS was 0.78 pmol/min in the presence of 50 bp double-stranded DNA. In contrast, in the presence of single-stranded SL2 DNA from HIV sstDNA, the production rate was 0.04 pmol/min, which was approximately 5% of that observed with the 50 bp double-stranded DNA (Figure 1 and Table 1; Supplementary Figure S1). These results demonstrate that the cGAS stimulatory activity of single-stranded SL2 DNA from HIV-1 is extremely low compared to that of the 50 bp double-stranded DNA (Figure 1 and Table 1).

**Table 1:** Production rates (*V*_p_) of 2′3′-cGAMP by cGAS in the presence of DNA

DNA	*V*_p_ (pmol/min)	*n*
50 bp double-stranded DNA	0.78 ± 0.21	5
Single-stranded SL2 DNA	0.04 ± 0.01	3
G3 Y-form DNA	0.59 ± 0.14	5

Averages and standard deviations are shown. The *n* values indicate the number of independent experiments.

**Figure 2:**
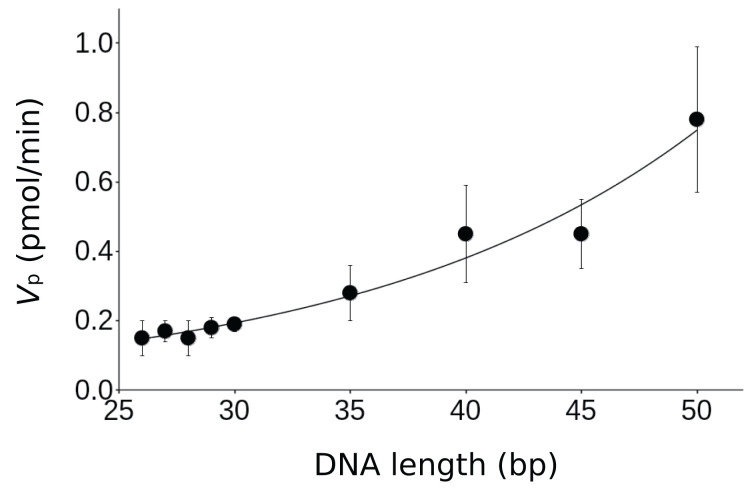
Dependence of 2′3′-cGAMP production rate on double-stranded DNA length At least three independent experiments were performed. Average and standard deviation values are shown in the graph.

Next, we compared the cGAS stimulatory activity of single-stranded SL2 DNA with that of double-stranded DNA shorter than 50 bp (Figure 2). The nucleotide sequences of the double-stranded DNA used in the experiments are listed in Supplementary Figure S1, with lengths ranging from 50 to 26 bp. The *V*_p_ value of single-stranded SL2 HIV-1 DNA was lower than that of the shortest 26 bp double-stranded DNA, which was 0.15 pmol/min (Figure 2 and Table 1). This corroborates that the cGAS stimulatory activity of SL2 HIV-1 DNA is remarkably low.

### cGAS stimulatory activity is reduced due to the guanine-to-cytosine mutation and the deletion of the unpaired guanosine trimers in G3 Y-form DNA

**Figure 3:**
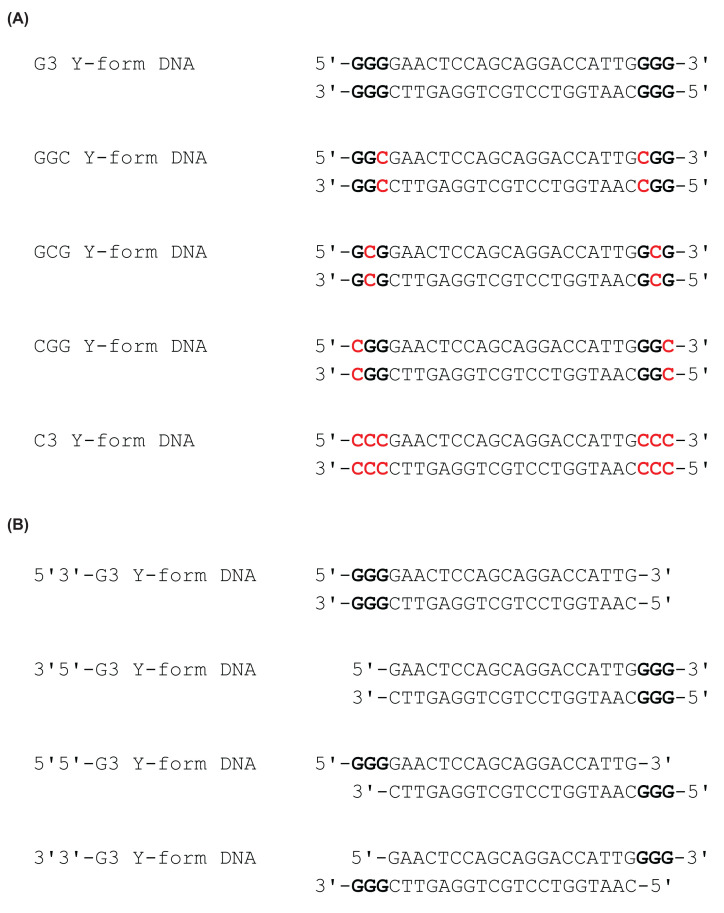
DNA sequences of G3-ended Y-form DNA and its mutants Unpaired guanosines and cytidines are highlighted in bold letters. (**A**) DNA sequences of G3 Y-form DNA, G-to-C mutants, and C3 Y-form DNA. The positions of cytidines are indicated in red font. (**B**) DNA sequences of deletion mutants of G3 Y-form DNA.

In the presence of G3 Y-form DNA, the production rate of 2′3′-cGAMP was 0.59 pmol/min, which was similar to that in the presence of 50 bp double-stranded DNA (Figure 3 and Table 1). G3 Y-form DNA consists of a 20 bp double-stranded region and four unpaired guanosine trimers at the 5′ and 3′ ends. We also measured the production rate of 2′3′-cGAMP in the presence of C3 Y-form DNA, in which guanosine trimers were replaced with cytidine trimers (Figure 3). The *V*_p_ value was 0.02 pmol/min in the presence of C3 Y-form DNA, which was similar to that of single-stranded SL2 HIV-1 DNA (Tables 1 and 2). We then investigated the effect of guanine-to-cytosine (G-to-C) mutations in the G3 Y-form DNA on cGAS enzymatic activity (Figure 3 and Table 2). The enzymatic activity of cGAS was remarkably reduced when cytidine was substituted for guanosine at any position within the guanosine trimer of the G3 Y-form DNA (Table 2). The cGAS stimulatory activity of the G-to-C mutants of the G3 Y-form DNA was similar to that of the C3 Y-form DNA and that of the single-stranded SL2 HIV-1 DNA (Figure 3, Tables 1 and 2). These results indicate that each guanosine within the unpaired guanosine trimer is necessary for the cGAS stimulatory activity of the G3 Y-form DNA.

**Table 2:** Production rates (*V*_p_) of 2′3′-cGAMP by cGAS in the presence of the G-to-C mutants of G3 Y-form DNA

DNA	*V*_p_ (pmol/min)	*n*
G3 Y-form DNA	0.59 ± 0.14	5
GGC Y-form DNA	0.04 ± 0.03	3
GCG Y-form DNA	0.03 ± 0.01	3
CGG Y-form DNA	0.03 ± 0.01	3
C3 Y-form DNA	0.02 ± 0.01	3

Averages and standard deviations are shown. The *n* values indicate the number of independent experiments.

We also examined the impact of the deletion of guanosine trimers from the G3 Y-form DNA on cGAS enzymatic activity (Figure 3 and Table 3). In the presence of these deletion mutants, the production rates of 2′3′-cGAMP were significantly reduced compared with those observed with intact G3 Y-form DNA (Table 3). Therefore, all four unpaired guanosine trimers are crucial for cGAS stimulatory activity.

**Table 3:** Production rates (*V*_p_) of 2′3′-cGAMP by cGAS in the presence of the deletion mutants of G3 Y-form DNA

DNA	*V*_p_ (pmol/min)	*n*
G3 Y-form DNA	0.59 ± 0.14	5
5′3′-G3 Y-form DNA	0.27 ± 0.05	3
3′5′-G3 Y-form DNA	0.13 ± 0.03	3
5′5′-G3 Y-form DNA	0.06 ± 0.01	3
3′3′-G3 Y-form DNA	0.03 ± 0.01	3

Averages and standard deviations are shown. The *n* values indicate the number of independent experiments.

### DNA binding to cGAS is weakened by the G-to-C mutation and the deletion of unpaired guanosine trimers

**Figure 4:**
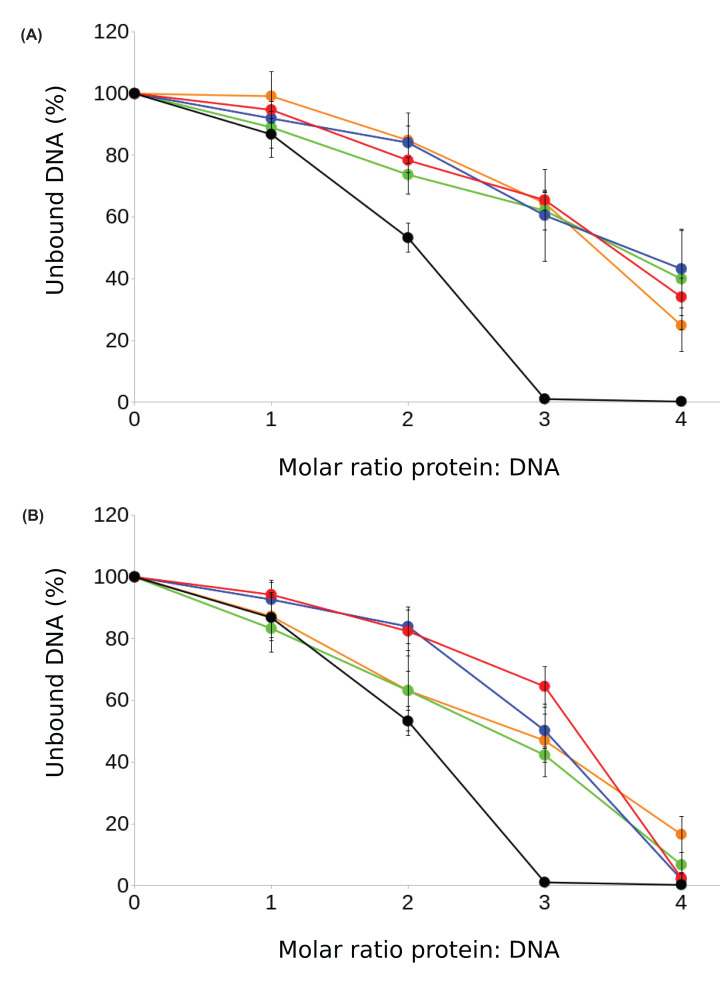
Percentages of unbound DNA at different cGAS/DNA ratios monitored by EMSA Three independent experiments were performed. Average and standard deviation values are shown in the graph. (**A**) Black: G3 Y-form DNA, red: GGC Y-form DNA, blue: GCG Y-form DNA, green: CGG Y-form DNA, orange: C3 Y-form DNA. (**B**) Black: G3 Y-form DNA, red: 5′3′-G3 Y-form DNA, blue: 3′5′-G3 Y-form DNA, green: 5′5′-G3 Y-form DNA, orange: 3′3′-G3 Y-form DNA.

We investigated the interaction between cGAS and G3 Y-form DNA using an electrophoretic mobility shift assay (EMSA). Unbound DNA was separated from the protein-DNA complex through electrophoresis, enabling us to determine the percentage of unbound DNA in the sample (Supplementary Figure S2). The percentage of unbound DNA was almost zero in the case that the cGAS/DNA ratio was 3 for G3 Y-form DNA (Figure 4). On the other hand, when the G-to-C mutants of the G3 Y-form DNA were used, the percentage of unbound DNA was more than 60% at the cGAS/DNA ratio of 3 (Figure 4). This indicates that the G-to-C mutation in unpaired guanosine trimers weakens the binding of Y-form DNA to cGAS. Additionally, EMSA revealed that the deletion of the unpaired guanosine trimers weakened the binding of cGAS and Y-form DNA (Figure 4).

## Discussion

To elucidate the mechanisms underlying cGAS-mediated type I interferon production in the innate immune response, it is important to detect cytokines or chemokines that are produced through the cGAS/STING signaling pathway. In previous studies, cells were transfected with DNA, and the induction of type I interferon by the stimulatory DNA was measured for this purpose [2,8,9,16,17]. This cell-based assay is sensitive for detecting cytokines or chemokines produced through the cGAS/STING signaling pathway. However, this assay may be influenced by variations in DNA transfection efficiency and the stability of DNA against deoxyribonuclease in the cytosol [16]. Furthermore, it’s worth noting that besides cGAS, other cytoplasmic DNA sensors may also influence the quantity of cytokines or chemokines produced following DNA transfection. Indeed, several cytoplasmic DNA sensors other than cGAS have been identified [1,18–24].

In the present study, we compared the cGAS stimulatory activity of DNAs by measuring the enzymatic activity of cGAS in the presence of DNA using HPLC. This approach enables the quantitative analysis of the amount of 2′3′-cGAMP produced by cGAS in the presence of DNA, making it suitable for comparing the cGAS stimulatory activity levels of different DNAs. The measurement of enzymatic activity is independent of DNA transfection efficiency, DNA stability against deoxyribonuclease, and other DNA sensors. Our analysis using HPLC can provide supplementary *in vitro* evidence by re-investigating previously established findings, without being affected by DNA transfection efficiency, DNA stability, and other DNA sensors.

This study compared the enzymatic activity of cGAS in the presence of three types of DNA. It is the first to report on a direct comparison of the production rate of 2′3′-cGAMP by cGAS in the presence of various DNA types. We used 50 bp double-stranded DNA derived from *Bacillus subtilis*, single-stranded SL2 DNA resulting from HIV-1 infection, and G3 Y-form DNA featuring four unpaired guanosine trimers. These specific DNA samples were chosen to represent diverse structures and origins, allowing us to explore the variations in cGAS stimulatory activity. Previous studies have shown that these DNAs induce type I interferon production by activating the cGAS/STING pathway [8,16]. Our study clearly demonstrated that cGAS was efficiently stimulated by a 50 bp double-stranded DNA and G3 Y-form DNA, consistent with previous findings [8,16]. Our results also indicated that each guanosine within the unpaired guanosine trimer was crucial for the cGAS stimulatory activity of G3 Y-form DNA. This is consistent with a previous study that demonstrated sequence-dependent interferon production by Y-form DNA [16]. Furthermore, we showed that the deletion of the unpaired guanosine trimers significantly reduced the binding of cGAS to Y-form DNA. The decrease in cGAS stimulatory activity could be explained by the weakening of the binding between cGAS and Y-form DNA.

Herzner et al. predicted that the 70-nucleotide single-stranded SL2 DNA forms a stem-loop structure comprising 21 base pairs in a stem sequence that is maximally 24–26 nucleotides in length, which makes it similar to a short double-stranded DNA [16]. This prediction aligns with our results, demonstrating that the production of 2′3′-cGAMP by cGAS in the presence of single-stranded SL2 HIV-1 DNA is remarkably low.

Our results showed that the cGAS stimulatory activity of the stem-loop-forming single-stranded SL2 DNA was very limited. The cGAS stimulatory activity of the stem-loop-forming SL2 HIV-1 DNA was lower than that of 26 bp double-stranded DNA and was comparable to those of the G-to-C mutants and the C3-ended Y-form DNA. On the other hand, a prior study [16] indicates that HIV SL2 can generate a certain level of type I interferon. The presence of single-stranded DNA in the cytosol during the early stages of HIV-1 infection could potentially engage multiple DNA sensors as well as cGAS, collectively contributing to the immune response against the virus. Additional investigation in an *in vivo* system or mouse models will be important to further investigate the role of cGAS recognition in the immune response against retroviruses, including HIV-1.

## Materials and methods

### Protein expression and purification

The expression plasmid of full-length human cGAS (residues 1-522) was constructed by inserting the DNA of cGAS into the plasmid pOPHLT-L [25]. The cGAS expression plasmid was used for the transformation of *Escherichia coli* SoluBL21 (Genlantis, San Diego, CA, U.S.A.). Human cGAS was expressed as a fusion protein of an N-terminal His6-tagged lipoyl domain and full-length cGAS [25]. The amino acid sequence of the TEV protease recognition site was inserted between the lipoyl domain and cGAS. After the optical density at 600 nm reached 0.4, protein expression was induced by adding an isopropyl β-D-thiogalactopyranoside to a final concentration of 0.5 mM. After cultivation of *E. coli* at 18°C for 18–20 h, the cells were harvested through centrifugation and resuspended in buffer A containing 20 mM Tris-HCl (pH 8.0 at 4°C), 500 mM NaCl, 10 mM imidazole, 10% glycerol, and 5 mM 2-mercaptoethanol. The cells were sonicated on ice, and supernatant was collected by centrifugation at 10200 ***g*** and 4°C for 60 min. The His6-tagged fusion protein of the lipoyl domain and cGAS was purified with a Ni-NTA resin equilibrated with buffer A. After eluting the His6-tagged protein from the Ni-NTA resin, TEV protease was added to the protein solution. This was followed by dialysis against buffer B containing 20 mM Tris-HCl (pH 8.0 at 4°C), 500 mM NaCl, 10% glycerol, and 5 mM 2-mercaptoethanol. The protein solution was passed through the Ni-NTA resin equilibrated with buffer B, and the flowthrough fraction was collected. The cGAS protein was further purified through gel-filtration chromatography on a Superdex 75pg HiLoad 16/60 column (GE Healthcare Bio-Sciences AB, Sweden) that had been pre-equilibrated with buffer C. This buffer contained 20 mM HEPES (pH 7.5), 250 mM KCl, 1 mM dithiothreitol, and 10% glycerol.

### Enzymatic activity assay

The enzymatic activity assay was conducted at 37°C in a total volume of 80 µl of 50 mM Tris-HCl (pH 7.5 at 25°C) containing 0.2 µM cGAS, 3 µM DNA, 50 µM ATP, 50 µM GTP, 100 mM NaCl, 5 mM MgCl_2_, and 0.1 mM ZnCl_2_. The DNAs were prepared by annealing corresponding oligonucleotides. After the enzyme reaction was initiated, an aliquot of the reaction mixture was taken at 0, 20, 40, and 60 min. The reaction mixture was incubated at 95°C for 5 min and then centrifuged. The supernatant (10 µl) was applied to high-performance liquid chromatography (HPLC). We used a HILIC column (4.6 mm × 250 mm, Cosmosil, Nacalai Tesque Inc., Kyoto, Japan) equilibrated with 60% methanol containing 8 mM sodium phosphate (pH 7.0) at a flow rate of 0.5 ml/min. The absorbance at 260 nm was used to detect 2′3′-cGAMP. The standard curve for estimating 2′3′-cGAMP concentrations was generated by plotting the peak areas of 2′3′-cGAMP in the HPLC chromatograms of different concentrations of 2′3′-cGAMP (InvivoGen, San Diego, CA, U.S.A.).

### Electrophoretic mobility shift assay (EMSA)

The DNAs were prepared by annealing corresponding oligonucleotides. DNA and cGAS were mixed in a final volume of 10 μl on ice for 30 min. The mixed samples contained 1 μM DNA, 20 mM Tris-HCl (pH 8.0 at 4°C), and 200 mM NaCl. The concentration of cGAS was set to 0–5 μM. TG0E buffer (25 mM Tris-HCl at pH 8.5, 190 mM glycine) was used as a gel running buffer. An 8% native polyacrylamide gel was prepared with TG0E buffer at pH 8.5, and was pre-run at 100 V for 10 min. Before applying samples, 2 μl of 50% glycerol was added to 10 μl of cGAS-DNA mixture. Samples (6 μl) were electrophoresed at 100 V in 8% TG0E gels. The gel was stained with ethidium bromide, and DNA was detected on a UV transilluminator. The band intensities were analyzed using ImageJ software (National Institutes of Health, Bethesda, MD, U.S.A.).

## Supplementary Material

Supplementary Figures S1-S2

## Data Availability

All data generated and analyzed in the present study are included in the manuscript and the supplementary files. Raw data are available on request.

## Abbreviations

AMP: adenosine monophosphate
ATP: adenosine triphosphate
Bp: base pairs
cGAMP: cyclic GMP-AMP
cGAS: cyclic GMP-AMP synthase
EMSA: electrophoretic mobility shift assay
G-to-C: guanine-to-cytosine
G3 Y-form DNA: Y-form DNA with unpaired guanosine trimers
GMP: guanosine monophosphate
GTP: guanosine triphosphate
HIV sstDNA: reverse transcription product of the first 181 nucleotides of the HIV-1 RNA genome
NTase: nucleotidyl transferase
STING: stimulator of interferon genes
*V*_p_: production rate
