# Supplementary material for: Enzymatic activity of cGAS in the presence of three types of DNAs: limited cGAS stimulation by single-stranded HIV-1 SL2 DNA
Source: Biosci Rep. 2024 Apr 4;44(4):BSR20240269. doi: 10.1042/BSR20240269 (PMC10994814; doi:10.1042/BSR20240269)
Supplement: Supplementary Figures S1-S2 [file BSR-2024-0269_supp.pdf]

Supplementary Figure 1

(A)

```
Double-stranded DNA

  50 bp DNA        5'-GGATACGTAACAACGCTTATGCATCGCCGCCGCTACATCCCTGAGCTGAC-3'
                   3'-CCTATGCATTGTTGCGAATACGTAGCGGCGGCGATGTAGGGACTCGACTG-5'

  45 bp DNA        5'-GGATACGTAACAACGCTTATGCATCGCCGCCGCTACATCCCTGAG-3'
                   3'-CCTATGCATTGTTGCGAATACGTAGCGGCGGCGATGTAGGGACTC-5'

  40 bp DNA        5'-GGATACGTAACAACGCTTATGCATCGCCGCCGCTACATCC-3'
                   3'-CCTATGCATTGTTGCGAATACGTAGCGGCGGCGATGTAGG-5'

  35 bp DNA        5'-GGATACGTAACAACGCTTATGCATCGCCGCCGCTA-3'
                   3'-CCTATGCATTGTTGCGAATACGTAGCGGCGGCGAT-5'

  30 bp DNA        5'-GGATACGTAACAACGCTTATGCATCGCCGC-3'
                   3'-CCTATGCATTGTTGCGAATACGTAGCGGCG-5'

  29 bp DNA        5'-GGATACGTAACAACGCTTATGCATCGCCG-3'
                   3'-CCTATGCATTGTTGCGAATACGTAGCGGC-5'

  28 bp DNA        5'-GGATACGTAACAACGCTTATGCATCGCC-3'
                   3'-CCTATGCATTGTTGCGAATACGTAGCGG-5'

  27 bp DNA        5'-GGATACGTAACAACGCTTATGCATCGC-3'
                   3'-CCTATGCATTGTTGCGAATACGTAGCG-5'

  26 bp DNA        5'-GGATACGTAACAACGCTTATGCATCG-3'
                   3'-CCTATGCATTGTTGCGAATACGTAGC-5'
```

(B)

```
70-nucleotide single-stranded SL2 DNA from HIV-1

  5'-CAGACGGGCACACACTACTTGAAGCACTCAAGGCAAGCTTTATTGAGGCTTAAGCAGTGGGTTCCCTAGT-3'
```

Supplementary Figure 1. DNA sequences of (A) double-stranded DNA and (B) 70-nucleotide single-stranded SL2 DNA from HIV-1.

Supplementary Figure 2

(A)

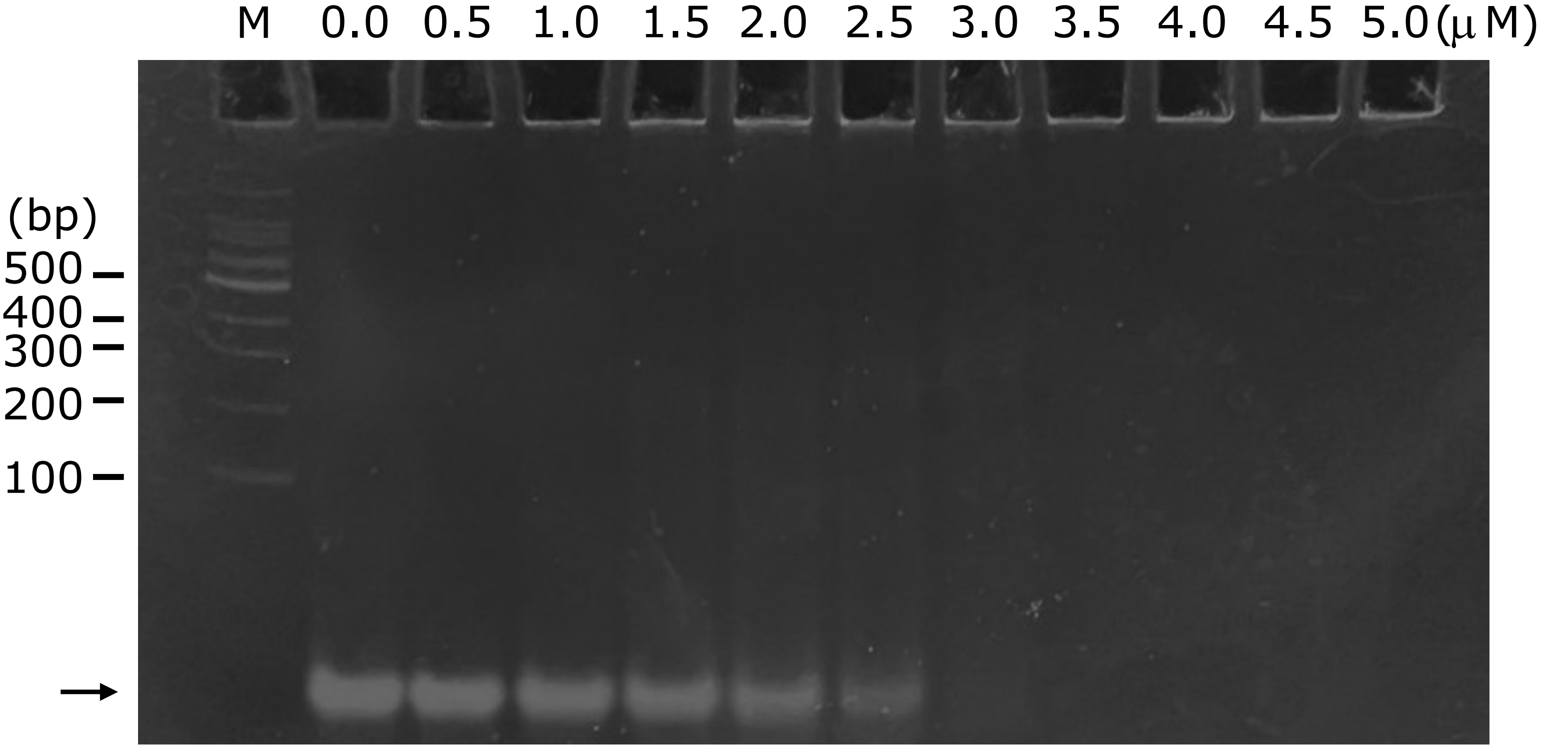

(B)

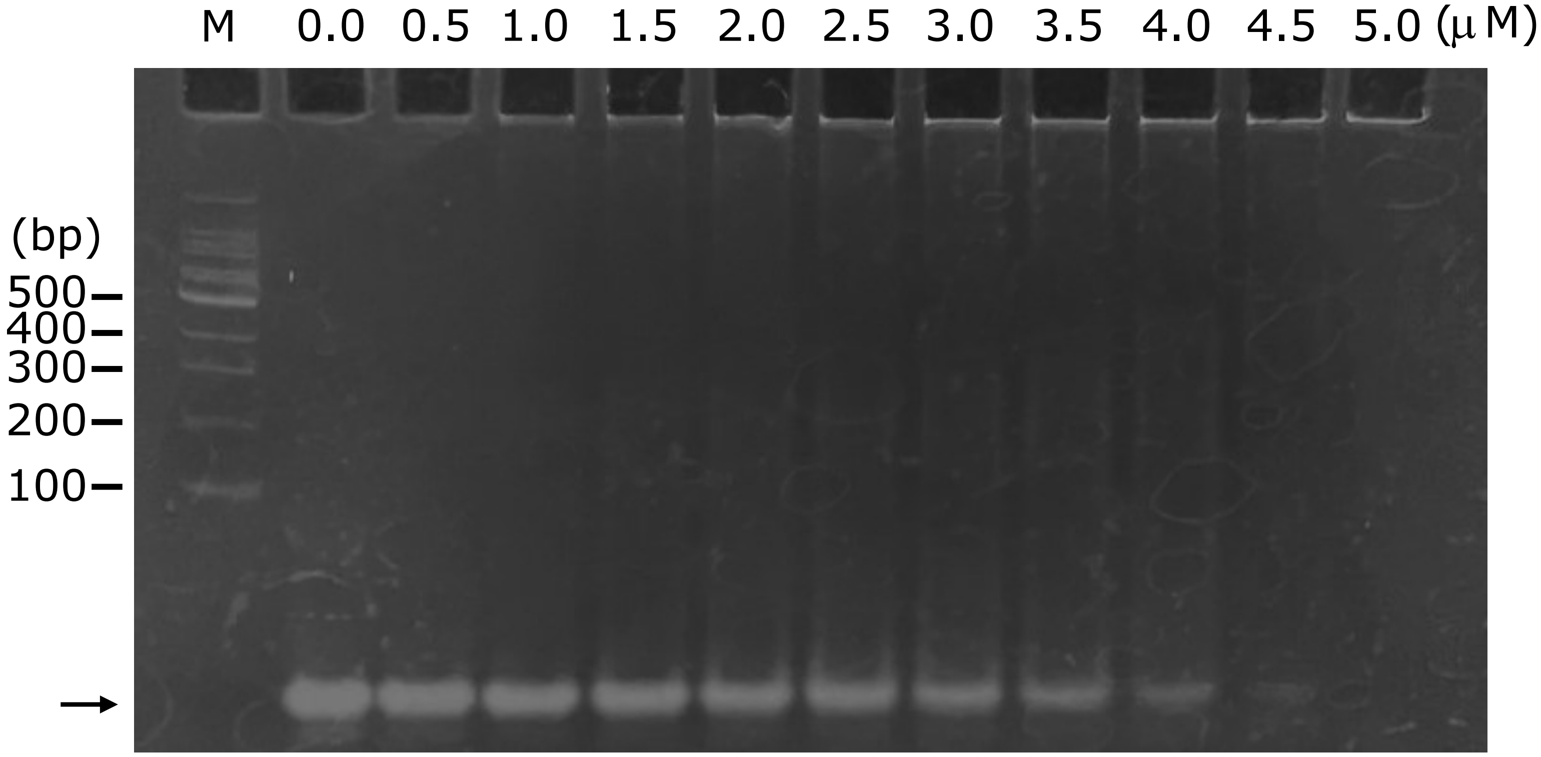

Supplementary Figure 2. EMSA assay of the binding of cGAS to DNA. The DNA concentration was set to be 1 μM. The concentration of cGAS was indicated at the top of each panel. The positions of unbound DNA are indicated by arrows. M refers to DNA markers with their size (in base pairs) indicated on the left. (A) G3 Y-form DNA. (B) C3 Y-form DNA.
